# Connection between Telomerase Activity in PBMC and Markers of Inflammation and Endothelial Dysfunction in Patients with Metabolic Syndrome

**DOI:** 10.1371/journal.pone.0035739

**Published:** 2012-04-25

**Authors:** Elias Rentoukas, Konstantinos Tsarouhas, Ioannis Kaplanis, Eleni Korou, Maria Nikolaou, George Marathonitis, Stavroula Kokkinou, Alexander Haliassos, Avgi Mamalaki, Demetrios Kouretas, Christina Tsitsimpikou

**Affiliations:** 1 Second Cardiology Department, Amalia Fleming General Hospital, Athens, Attiki, Greece; 2 Cardiology Division, General Hospital of Karditsa, Terma Tavropou, Karditsa, Greece; 3 A' Pathology Department, Amalia Fleming General Hospital, Athens, Attiki, Greece; 4 Cytogenetic Unit, Sismanoglio General Hospital, Athens, Attiki, Greece; 5 ESEAP - Greek Proficiency testing scheme (for Laboratory Medicine), Diamedica Laboratories Department SA, Athens, Attiki, Greece; 6 Department of Biochemistry, Hellenic Pasteur Institute, Ampelokipi, Athens, Greece; 7 Department of Biochemistry and Biotechnology, University of Thessaly, Larisa, Greece; 8 General Chemical State Laboratory of Greece, Athens, Attiki, Greece; University of Tor Vergata, Italy

## Abstract

Metabolic syndrome (MS) is a constellation of metabolic derangements associated with vascular endothelial dysfunction and oxidative stress and is widely regarded as an inflammatory condition, accompanied by an increased risk for cardiovascular disease. The present study tried to investigate the implications of telomerase activity with inflammation and impaired endothelial function in patients with metabolic syndrome. Telomerase activity in circulating peripheral blood mononuclear cells (PBMC), TNF-α, IL-6 and ADMA were monitored in 39 patients with MS and 20 age and sex-matched healthy volunteers. Telomerase activity in PBMC, TNF-α, IL-6 and ADMA were all significantly elevated in patients with MS compared to healthy volunteers. PBMC telomerase was negatively correlated with HDL and positively correlated with ADMA, while no association between TNF-α and IL-6 was observed. IL-6 was increasing with increasing systolic pressure both in the patients with MS and in the healthy volunteers, while smoking and diabetes were positively correlated with IL-6 only in the patients' group. In conclusion, in patients with MS characterised by a strong dyslipidemic profile and low diabetes prevalence, significant telomerase activity was detected in circulating PBMC, along with elevated markers of inflammation and endothelial dysfunction. These findings suggest a prolonged activity of inflammatory cells in the studied state of this metabolic disorder that could represent a contributory pathway in the pathogenesis of atherosclerosis.

## Introduction

Metabolic syndrome (MS) is a constellation of metabolic derangements commonly coexisting in the same patient that has reached epidemic proportions [1]. A cluster of cardiovascular and pro-thrombotic risk factors, such as insulin resistance, impaired glucose tolerance, dyslipidemia, obesity and elevated blood pressure characterize MS. MS is reported to be associated with vascular endothelial dysfunction and oxidative internal milieu [2] and is widely regarded as an inflammatory condition [3], [4], accompanied by an increased risk for cardiovascular disease [5]. MS represents a combination of synergistic vascular pathologies that lead to an accelerated atherogenic state that compromises the ability of the patient to satisfactorily respond to humoral, cellular, and mechanical stresses.

Endothelial dysfunction with impaired nitric oxide (NO) bioavailability has been implicated in insulin resistance and hypertension. Asymmetric dimethyl arginine (ADMA) is linked to the impairment of endothelial, NO-dependent, function by inhibiting endogenous nitric oxide synthetase in a concentration-dependent manner, and has been associated with cardiovascular diseases [6], [7], [8].

Telomerase activity is crucial for preservation of telomeres' function and structure [9]. Telomeres, the ends of eukaryotic chromosomes, are structures strictly related to the phenomenon of cellular senescence [10]. Cellular senescence occurs after an extended period of cell divisions, but it can occur earlier in case the cell is exposed to various types of stresses, oxidative stress being one of them [9]. Telomerase activity may also be interrelated to inflammation, especially in cell types like peripheral blood mononuclear cells (PBMC) implicated in this process [11]. Soluble CD163 (sCD163) represents a monocyte/macrophage-specific scavenger receptor cleaved under the effect of inflammatory stimuli and subsequently released systemically from monocyte/macrophage cell membranes, suggesting that sCD163 is a marker of monocyte/macrophage activation [12].

The present study tried to investigate the implications of telomerase activity with inflammation and impaired endothelial function in patients with metabolic syndrome.

## Methods

### Participants

Patients were classified based on the presence or absence of MS at baseline using the AHA/NHLBI definition [13]: triglycerides ≥150 mg/dL; high-density lipoprotein cholesterol (HDL) *<*40 mg/dL in males or *<*50 mg/dL for females; blood pressure (BP) ≥130/85 mm Hg or treatment with antihypertensive medications; and fasting blood glucose ≥100 mg/dL or treatment with oral hypoglycaemic drugs or insulin injection; waist circumference ≥102 cm (men), ≥88 cm (women). Patients who had 3 of 5 criteria were regarded as having MS. Thirty nine (39) patients with MS treated on a regular basis at the “Amalia Fleming" General Hospital in Athens, Greece, volunteered to participate in the study. Twenty (20) age and sex-matched healthy volunteers were also used as reference for normal population values of TNF-α, IL-6, ADMA and telomerase activity that were monitored.

### Description of Procedures or Investigations undertaken

In the morning and after an at-least-30-min supine rest, non-fasting venous blood samples were drawn from all patients and healthy volunteers, centrifugated within 30 min and stored at −20°C. Serum samples were later analysed for ADMA using an ADMA-ELISA kit (DLD) (sensitivity 0.05 µmol/l, upper limit of the working range 5.0 µmol/l, mean intra-assay variation 6.05%). TNF-α and IL-6 were measured using the IMMULITE® 1000TNF-α (sensitivity 1.7 pg/ml, upper limit of the working range 1000 pg/ml, mean intra-assay variation 3.2%) and IMMULITE® 1000IL-6 (sensitivity 2 pg/ml, upper limit of the working range 1000 pg/ml, mean intra-assay variation 4.65%) assays (Siemens). sCD163 was determined using an ELISA kit from Trillium Diagnostics as previously described [12]. The method for detection of telomerase activity and isolation of PBMC is described elsewhere [14]. Briefly, telomerase activity in PBMC was measured using a commercial telomerase PCR-ELISA (Roche Diagnostics Corp., Indianapolis, IN, USA), based on the telomeric repeat amplification protocol [15].

### Ethics

Written informed consents were obtained from all participants. The research ethics committee of the “Amalia Fleming" Hospital approved the procedures. The Declaration of Helsinki (2000) and the applicable national standards as they relate to the involvement of human subjects in research were enforced. No external funding was received for this study.

### Statistical methods

All results are presented as mean values ± SD. Statistical analyses were performed with SPSS version 14 (SPSS Inc., Chicago, IL, USA). Significant differences between means for the same parameters were investigated with repeated measures ANOVA and paired *t*-test analyses. Independent *t-*tests were used to compare mean values between groups. Pearson and Spearman correlations and linear regression analysis was conducted to investigate associations between various variables. Differences between categorical variables were assessed by the chi-square test. Multiple linear regression analyses after log transformation of the dependent variable, since the distribution was skewed, were performed to evaluate the relationship between PBMC telomerase activity and factors associated with the development of cardiovascular risk (hypertension, diabetes, smoking, hyperlipidemia, waist circumference). Multiple linear regression analyses with backward selection was applied to investigate the correlation between PBMC telomerase activity and ADMA. A list of possible confounders (hypertension, diabetes, smoking, hyperlipidemia, waist circumference) was included in the initial variables set. A *p*-value *≤*0.05 was considered statistically significant.

## Results

Thirty-nine patients (mean age 54±9.9 years) were included in the study. Table 1 summarizes the demographic, clinical and epidemiological characteristics of patients and healthy volunteers and values for all biochemical parameters monitored. The MS patients of this study are characterised by disturbed lipidemic profile (92.1% elevated TG, 89,4% low HDL) and obesity (100% abnormal waist circumference), while the prevalence of diabetes was 38.5% and of hypertension 52.6%.

**Table 1:** Demographic and clinical characteristics and biochemical parameters monitored in the patients of the study group and the healthy individuals that provided reference values for all biochemical parameters (healthy controls).

Demographic and Clinical Characteristics		Healthy controls	Patients with metabolic syndrome	P^a^
Gender	Male	15	29	0.911
	Female	5	9	
Age (years)		56±10 (31–60)	54±9.9 (33–69)	0.723
Waist circumference (cm)		114±17.6 (78–135)	129±15.8 (105–160)	0.002
Hypertension (mmHg)	YES	2 (90–145)	20 (92–145)	0.001
	NO	18 (68–125)	18 (70–122)	
Diabetes Mellitus (DB)	YES	0	16	0.001
	NO	20	22	
Triglycerides (TG) (mg/dl)^b^		127±52.4 (46.1–230)	259±72.0 (138–422)	0.000
LDL (mg/dl)		130±24.3 (80.2–177)	147±35.0 (76.2–250)	0.220
HDL (mg/dl)		47.1±8.61 (24.1–59.7)	43.4±9.91 (26.2–64.1)	0.037
Smoking		9	16	0.832
Family history of Coronary Disease		5	10	0.933
Chronic kidney failure		0	1	-
General surgical operations		1	10	-
ACEI*^3^* administration		0	10	-
Statins administration		0	8	-
Vascular disease		0	0	-
TNF-α (pg/ml)		9.18±3.93	35.6±24.5	0.003
IL-6 (pg/ml)		8.89±3.34	34.9±24.8	0.006
ADMA (µmol/ml)		1.15±0.43	2.07±0.67	0.014
Telomerase activity (OD)*^4^*		0.67±0.28	1.67±0.60	0.002
sCD163 (ng/ml)		534±164	876±301	0.032

*Values are means*±*SD.*

a *Comparison between Patients with metabolic syndrome and Healthy controls.*

b *Normal values or range of values for general population: TG 0–160 mg/dl, LDL optimal >100 mg/dl near optimal 100–1129 mg/dl above optimal 130–159 mg/dl borderline high 160–189 mg/dl high >190 mg/dl*, *HDL* >55 *mg/dl* (male), >65 *mg/dl* (female).

*LDL: low–density lipoprotein cholesterol; HDL: high–density lipoprotein cholesterol; TNF-α: Tumor necrosis factor α; ACEI: angiotensin converting enzyme inhibitor; OD: Optical Density.*

Telomerase activity in PBMC, TNF-α, IL-6, sCD163 and ADMA were all significantly elevated in patients with MS compared to healthy volunteers.

Upon analysis of all patients with MS for factors associated with the development of cardiovascular risk by multiple linear regression, significant negative correlations were only found between PBMC telomerase activity and HDL (r = −0.653, *p* = 0.021 and waist circumference (r = −0.621, *p* = 0.031). PBMC telomerase activity was also positively correlated with ADMA (r = 0.604, *p* = 0.038) and sCD163 (r = 0.556, *p* = 0.044), while no association between TNF-α and IL-6 and PBMC telomerase activity was observed. sCD163 were nearly significantly correlated with ADMA (r = 0.445, *p* = 0.058) and TNF-α (r = 0.301, *p* = 0.064).

TNF-α was found statistically increased in females compared to males only in the patients group. Telomerase activity, IL-6 and ADMA levels did not differ with sex. It should be noted, though, that the average age of women enrolled in the patients' group (51.7±5.45 years) was statistically higher (p = 0.022) compared to the healthy volunteers (35.6±5.18 years) and did not differ from the average age of male patients (54.5±10.9 years). Women above the average age of menopause (51 years) [16] are losing with increasing age the anti-inflammatory and vasoprotective effects of estrogens [17]. Waist circumference was again negatively correlated to TNF-α (r = 0.613, *p* = 0.019).

IL-6 was increasing with increasing systolic pressure both in the patients with MS and in the healthy volunteers (r = 0.354, *p* = 0.047), while smoking and diabetes were positively correlated with IL-6 only in the patients' group.

## Discussion

To the authors' knowledge, this is the first study to report on elevated telomerase activity in PBMC of patients with MS. Previous studies on haemodialysis and diabetic patients [18], [19] and patients with rheumatoid arthritis [20] have found lower PBMC telomerase activity suggesting that in a cell type like PBMC, which is directly implicated in the inflammatory process, it is generally expected a pre-mature senescence for a higher inflammatory activity [21]. On the other hand, PBMC telomerase levels measured in our study are comparable with those recently reported for overweight African Americans [22]. Furthermore, MS may be an important contributory factor for coronary artery disease through increased oxidative stress and induces subclinical atherosclerosis [2]. In that sense our results coincide with those reported on elevated polymorphonuclear neutrophils (PMN) telomerase in patients with unstable angina [23] and suggest a systemic activation of blood cells of the immune system. Garlichs et al. [24] have recently observed a marked delay of circulating PMN apoptosis in patients with acute coronary syndromes. Activated PBMC telomerase in MS patients could represent a consistent and persisting inflammation state, as neutrophil apoptosis has been identified to be one of the key mechanism to switch off inflammation [23]. In the same line of evidence, Gizard et al. found activated telomerase in macrophages [25].

Recently, a significant role in the regulation of the immune response accompanying atherosclerosis has been ascribed to interactions between activated T cells, promoting the expression of systemic inflammatory response factors participating in atherogenesis, such as TNF–α and IL-6 [26], [27]. Both TNF-α and IL-6 were found elevated in the MS patients of the present study, which is in accordance with the vascular biology described for MS [5]. TNF-α doesn't seem to correlate either with insulin resistance or with endothelial dysfunction in men with MS [28]. In the present study no association between TNF-α and IL-6 and PBMC telomerase activity was observed probably due to different and multi-factorial underlying activation mechanisms possibly involving endothelial damage and oxidative stress. Furthermore, systemic cytokines levels may underestimate local inflammation.

The elevation of sCD163 reported in our MS patients provides new information that the increased telomerase activity in PMBCs coexists with monocyte/macrophage activation. A recent study has revealed that monocyte/macrophage activation, as reflected by sCD163 levels, is strongly associated with HOMA-IR in normal-weight and obese subjects and thus may be an important determinant of insulin resistance in obesity [12].

Numerous metabolic abnormalities found in the metabolic syndrome cause an endothelial cell dysfunction by affecting NO synthesis or degradation [29]. There is evidence that NO possesses anti-inflammatory and anti-atherosclerotic properties [30]. ADMA increases in MS [6], although ethnic-specific or environmental differences may influence its levels [31]. We currently report strong positive correlation of PBMC telomerase activity with elevated serum ADMA in the MS patients of the present study. An ADMA-induced cycle of PMN activation has recently been reported [32]. Furthermore, endothelial impairment and activation of telomerase are both enhanced through the NF-κB transcription factor action: endothelial adhesion molecules are expressed in mononuclears in atherosclerotic lessions, with the current mediation of TNF-α pro-inflammatory effect [33] and the expression of the catalytic subunit telomerase reverse transcriptase (TERT) is induced in macrophage [25]. The nearly significant correlation of elevated sCD163 with ADMA and TNF-α in our MS patients could support the view that endothelial dysfunction through mediated TNF-α mechanisms lies behind the reported prolonged PBMC life cycle. In line with this conclusion, Satoh et al. reported significant shortening of telomere length, correlated with oxidative DNA damage, in endothelial progenitor cells (EPC) of patients suffering from coronary artery disease (CAD), this being even more intense in CAD patients with MS, inducing endothelial cell senescence and dysfunction [34]. Damage to the endothelium may thus be the key factor in the promotion of the atherogenic and inflammatory process in MS.

The high anti-oxidant and anti-inflammatory activities of HDL, which are associated with protection from cardiovascular disease [35] have become evident in the present study through the negative correlation found with PBMC telomerase activity. The association of telomerase activity with anthropometric measures, such as waist circumference and BMI, remains unclear in the literature [36], [37] and the negative correlations of waist circumference with PBMC telomerase activity and TNF-α observed in our MS patients needs to be further elucidated. Cardiovascular risk factors, such as smoking and hypertension don't correlate with PBMC telomerase activity, in agreement with previously published data [23], but strongly affect IL-6 in the present study.

In conclusion, in patients with MS with a strong dyslipidemic profile and low diabetes prevalence, significant telomerase activity was detected in the circulating PBMC, along with elevated markers of inflammation and endothelial dysfunction. These findings suggest a prolonged activity of inflammatory cells in the studied state of this metabolic disorder that could represent a contributory pathway in the pathogenesis of atherosclerosis. Further studies are warranted in order to establish the precise prognostic value of telomerase reactivation in MS.
